# A graph-based approach to variant description extraction from sequences

**DOI:** 10.1093/nargab/lqaf173

**Published:** 2025-12-08

**Authors:** Mark A Santcroos, Walter A Kosters, Mihai Lefter, Jeroen F J Laros, Jonathan K Vis

**Affiliations:** Department of Human Genetics, Leiden University Medical Center, 2333 ZC Leiden, the Netherlands; Department of Clinical Genetics, Leiden University Medical Center, 2333 ZC Leiden, the Netherlands; Leiden Institute of Advanced Computer Science, Leiden University, 2333 CC Leiden, the Netherlands; Department of Human Genetics, Leiden University Medical Center, 2333 ZC Leiden, the Netherlands; Department of Human Genetics, Leiden University Medical Center, 2333 ZC Leiden, the Netherlands; National Institute for Public Health and the Environment (RIVM), 3721 MA Bilthoven, the Netherlands; Department of Human Genetics, Leiden University Medical Center, 2333 ZC Leiden, the Netherlands; Leiden Institute of Advanced Computer Science, Leiden University, 2333 CC Leiden, the Netherlands

## Abstract

Accurate variant descriptions are of paramount importance in the field of genomics. The domain is confronted with increasingly complex variants, e.g. combinations of multiple indels, making it challenging to generate proper variant descriptions directly from chromosomal sequences. We present a graph based on all minimal alignments that is a complete representation of a variant, which gives insight into the nature of a variant compared to a single variant description. We provide three complementary extraction methods to derive variant descriptions from this graph, including one that yields domain-specific constructs from the HGVS nomenclature. Our experiments show that our methods in comparison with dbSNP, the authoritative variant database from the NCBI, result in identical HGVS descriptions for simple variants and more meaningful descriptions for complex variants, in particular for repeat expansions and contractions.

## Introduction

Insights into a person’s DNA are of relevance for a wide range of applications, not limited to clinical and research settings. Sequencing is the process to obtain the genetic sequence from DNA. Typically, this results in segments of chromosomes that need to be aligned to a reference genome, e.g. Genome Reference Consortium Human Build 38 (GRCh38) for human samples, in order to reconstruct the individual’s complete chromosomal sequences.

Because the human genome is large (circa $3 \cdot 10^9$ nucleotides), and the individual differences between two genomes are relatively small (circa $ 0.6 \% $ [1]), it is practical to only look at the differences. These differences are called genetic variants. In practice, these are recorded by most variant calling tools in the Variant Call Format (VCF) [2]. Variants appear in various formats in clinical communication and scientific literature, and are also stored in databases. The effectiveness of this information exchange largely depends on the specific format and quality of how these variants are described [3, 4].

The Human Genome Variation Society (HGVS) Variant Nomenclature Committee [5] publishes recommendations [6] for HGVS variant descriptions, that are intended to be human-readable, but provides no guidelines on how to construct them from sequences. A standardization effort with a machine-readable focus is the Variant Representation Specification (VRS) [7]. SPDI features a single replacement notation (Sequence, Position, Deletion, Insertion) and employs the Variant Overprecision Correction Algorithm (VOCA) for normalization. The Single Nucleotide Polymorphism Database (dbSNP) [8] is a public archive for genetic variation made available by National Center for Biotechnology Information (NCBI). The widespread usage of the dbSNP database makes their specific choice in formatting of HGVS variants quite prevalent.

In [9], significant inconsistencies in variant descriptions across tools and databases are observed, noting that it specifically hinders automatic lookup. Often variant description languages define a *normalization* procedure to select one universally accepted description from a set of possibilities. Beyond normalization, in [10] the authors also discuss many other challenges to effectively curate variants from literature. The authors of [11] show that searching for a single description in PubMed does not retrieve all relevant articles for a variant. In a more clinical study [12] the authors observe that differences in nomenclature can “hide clinically relevant information in plain sight.” Challenges with VCF normalization have been addressed in [13, 14]. In [15] the authors observe that in repetitive genomic regions, different descriptions of the same deletions can lead to the same result. A sequence-based normalization method specifically for concise HGVS descriptions is discussed in [16]. The problem of choosing an accurate variant description and assisting the user with variant normalization has given rise to many platforms [17–20].

**Figure 1.**
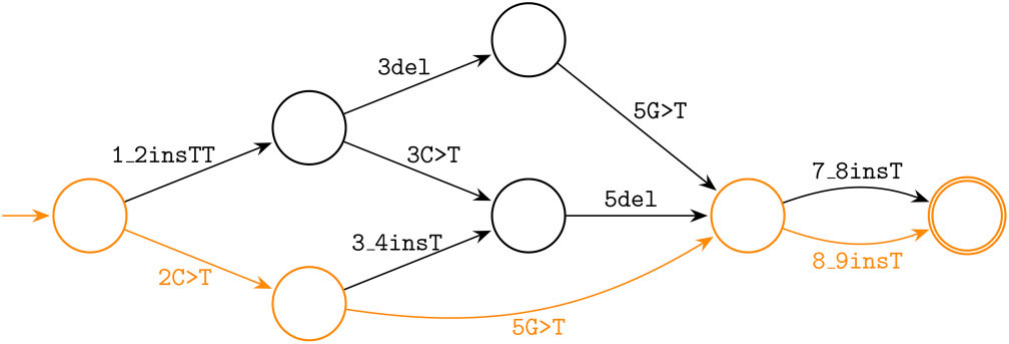
A graph containing all LCS alignments for the strings ACCTGACT and ATCTTACTT. The paths through the graph correspond with six HGVS descriptions. The highlighted path with the description [2C>T;5G>T;8_9insT] corresponds to the HGVS recommendations.

In the remainder of this paper we focus on a formal approach to calculate variant descriptions. Central is the problem of computing the distance between two strings and obtaining a series of edit operations [21]. Instead of focusing on *one* series of edit operations, we consider *all* minimal alignments corresponding to the simple edit distance [22] to generate variant descriptions. We introduce a new type of graph that is constructed from all these minimal alignments; a simplified example visualization using HGVS notation is shown in Fig. 1. Based on the structure of this graph, we provide three complementary methods to derive variant descriptions. We experimentally assess the effectiveness of our method by comparing our results to the HGVS descriptions in dbSNP. Finally, we discuss the broader implications of our methods and provide forward-looking statements.

## Materials and methods

We start with preliminary definitions that we build upon later. Let $\Sigma$ be a non-empty finite *alphabet* of *symbols*, e.g. $\Sigma = \lbrace \mathtt {A}, \mathtt {C}, \mathtt {G}, \mathtt {T}\rbrace$, and $S$ a finite *string* over $\Sigma$. The *length* of string $S$, written as $|S|$, is the number of symbols in $S$. The empty string (without any symbols, also said with length 0) is denoted as $\varepsilon$. We refer to the symbol at position $i$ of $S$ as $S_i$, with $0 \le i < |S|$. We use the natural extension of this notation to refer to *substrings* of $S$, i.e., $S_{i\ldots j}$ is the string containing the contiguous symbols $S_i,\ldots ,S_{j-1}$, with $0 \le i \le j \le |S|$. Note that $S_{i\ldots i} = \varepsilon$. String $U$ is a *subsequence* of string $S$ if $U = \varepsilon$ or there exists a sequence of integers $i_1,\ldots ,i_{|U|}$ such that $0 \le i_1 < \ldots < i_{|U|} < |S|$ and $U = S_{i_1}\ldots S_{i_{|U|}}$.

To describe differences between two strings $R$ (called *reference* sequence) and $O$ (called *observed* sequence) we introduce the *replacement* (also known as substitution) operator $i\!:\!j\, /\, S$, with $0 \le i \le j \le |R|$ and $S$ a substring of $O$ that replaces the symbols $R_i\ldots R_{j - 1}$ with the string $S$. When $i = j$, the string $S$ is inserted immediately before (the symbol on) the $i$-th position. We denote the empty replacement as $\lambda$. The string $R$ can be transformed into $O$ by applying a series of replacement operations.

A *longest common subsequence* (LCS) for two strings $R$ and $O$ is a longest string $U$ such that $U$ is a subsequence of both $R$ and $O$. In general, many of such LCSs exist for a given $R$ and $O$. Often, only the length of the LCSs is of interest. The classic approach to calculate the (length of the) LCSs is to solve the well-known recurrence relation using dynamic programming to fill a table row by row [21].

### All LCS alignments

We are interested in finding all LCS alignments for a pair of highly similar strings. It is well-known [23] that LCS alignment is related to calculating the *simple edit distance*; see, e.g. [22]. For only calculating the simple edit distance, the OND algorithm [24] and its later refinement the ONP algorithm [25] are considered practical methods in both space and time complexity [23]. However, in general, these methods do not yield all minimal alignments. In this section we show that a straightforward extension of the ONP algorithm does yield all minimal alignments, providing us with an elegant solution to the problem.

Contrary to the classic dynamic programming approach that computes a table row by row, here the calculation iteratively expands along the diagonals of the table. The first iteration starts with a set of numbered diagonals $\mathit {FP} = \lbrace 0, \ldots , |R| - |O|\rbrace$, where 0 refers to the main diagonal. Each iteration, the diagonals in $\mathit {FP}$ are processed outside in, finishing with the $|R| - |O|$ diagonal. Every diagonal is expanded to the maximum of the furthest points of its two flanking diagonals and possibly further as long as symbols from the two strings match. As long as the bottom-right of the table is not reached, we add the two flanking diagonals to the set $\mathit {FP}$ for the next iteration. The computed elements are never stored, only keeping track of the contour of the computed elements by storing the farthest position on the diagonals is required.

The length of the LCSs for string $R$ and string $O$ at position $(i, j)$, with $0 \le i < |R|$ and $0 \le j < |O|$, can be defined as:


(1) \begin{eqnarray*}
&& |\text{LCS}(R_{0\ldots i}, O_{0\ldots j})| = \\ && \left\lfloor \frac{i + j - ||R| - |O|| - 2p + |(|R| - i) - (|O| - j)|}{2}\right\rfloor + 1,\\
\end{eqnarray*}

for the $p$-th iteration from the ONP algorithm.

**Figure 2.**
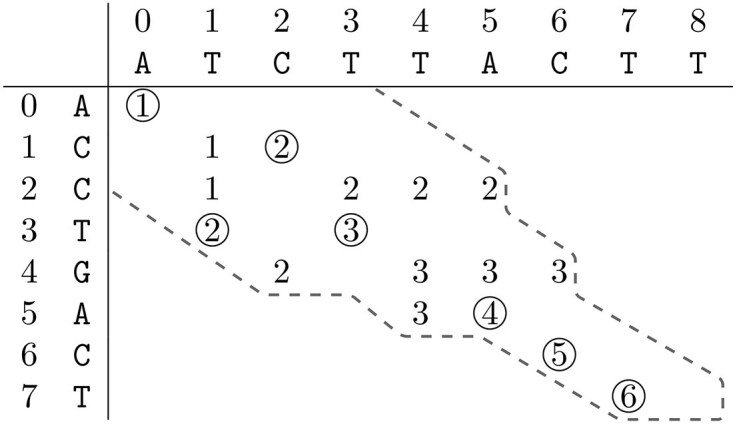
The computed elements by the original ONP algorithm with their values according to Equation 1 for $R = \mathtt {ACCTGACT}$ and $O = \mathtt {ATCTTACTT}$. The dashed line marks the contour of the solution space, the final set $\mathit {FP}$. Matching symbols are circled.

**Figure 3.**
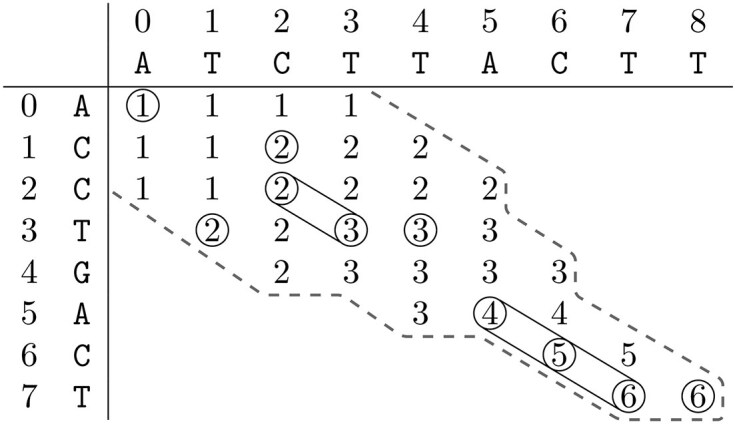
The computed elements of our extension of the ONP algorithm with their values according to Equation 1 for $R = \mathtt {ACCTGACT}$ and $O = \mathtt {ATCTTACTT}$. Consecutive matches are grouped.

Figure 2 depicts the computed elements by the ONP algorithm for $R = \mathtt {ACCTGACT}$ and $O = \mathtt {ATCTTACTT}$. We observe that the contour corresponds to the frontier of the A* implementation in [22]. Not all elements in the contour are calculated, including some of the matches. In order to calculate all LCS alignments, we require all elements with matching symbols within the contour. The matching T at $(3, 4)$ is a concrete example of a match that the ONP algorithm does not compute, but that we require. Therefore, instead of just taking the maximum position of the adjacent diagonals, we have to check for matching symbols up to the maximum, resulting in Fig. 3.

As we are interested in all LCS alignments, we also require an efficient method for storing all the matches. Given the context of highly similar sequences, we record all matches into groups of consecutive matches, represented by a triple $(i, j, \ell )$, where $i$ and $j$ are the coordinates of the positions of the first match together with $\ell$, the number of consecutive matches, effectively limiting the number of entries. The triples are stored in an *LCS-nodes* data structure indexed on the zero-based string position in an LCS of the last match of the node. Matches $(0, 0)$, $(1, 2)$, $(2, 2)$, $(3, 1)$, $(3, 3)$, $(3, 4)$, $(5, 5)$, $(6, 6)$, $(7, 7)$, $(7, 8)$ from Fig. 3 are thus stored as:


\begin{eqnarray*}
0&&: (0, 0, 1) \\1&&: (1, 2, 1), (3, 1, 1) \\2&&: (2, 2, 2), (3, 4, 1) \\3&&: \lambda \\4&&: \lambda \\5&&: (5, 5, 3), (7, 8, 1) ,
\end{eqnarray*}

where $\lambda$ denotes the empty list. The number of consecutive matches $\ell$ is also referred to as the length of an element.

The ONP algorithm swaps $R$ and $O$ when $|R| < |O|$ as it explores the problem from the perspective of deletions. It can freely do so as it only calculates the distance. As we compute the edit operations from $R$ to $O$, our extension takes care of not having to swap the strings.

### Constructing the compressed LCS-graph

**Figure 4.**
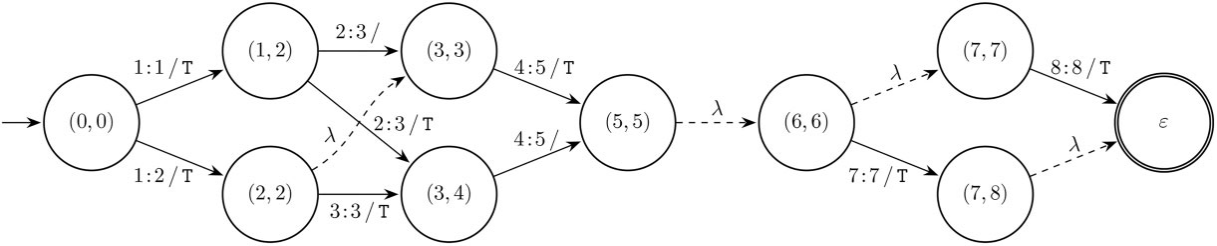
LCS-graph of $R = \mathtt {ACCTGACT}$ and $O = \mathtt {ATCTTACTT}$. Nodes represent matches and are labeled with their respective positions $(i, j)$. Edges describe the minimal replacements between the nodes. Dashed edges represent empty replacements. The leading edge indicates the source node and the double-circled node indicates the sink node.

The LCS-graph presented in [22, 26] is a directed acyclic graph that consists of nodes representing single symbol matches for all LCSs. These graphs always have a single source node and a single sink node. Labeled edges connect nodes for consecutive symbols in an LCS. Figure 4 shows the LCS-graph for the earlier discussed example.

**Figure 5.**
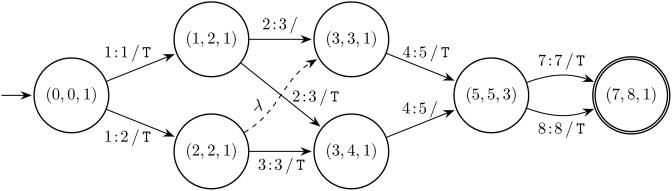
Compressed LCS-graph of $R = \mathtt {ACCTGACT}$ and $O = \mathtt {ATCTTACTT}$. Nodes represent matches and are labeled as $(i, j, \ell )$. Edges describe the minimal replacements between the nodes. Multiple edges representing unique minimal replacements between two nodes can exist.

In this section we introduce the *compressed LCS-graph* (cLCS-graph) that is a directed acyclic multigraph constructed from the (consecutive) matches in the LCS-nodes data structure. Figure 5 shows the cLCS-graph for the earlier discussed example.

We say that node $(i, j, k)$ is *dominated* by node $(i^{\prime }, j^{\prime }, k^{\prime })$ if $i + k < i^{\prime } + k^{\prime }$ and $j + k < j^{\prime } + k^{\prime }$. There is an edge between node $(i, j, \ell )$ and node $(i^{\prime }, j^{\prime }, \ell ^{\prime })$ for every $0 \le k < \ell$ and every $0 \le k^{\prime } < \ell ^{\prime }$ such that the following two conditions are met: node $(i, j, k)$ is dominated by node $(i^{\prime }, j^{\prime }, k^{\prime })$ and they are consecutive matches in an LCS: $|\text{LCS}(R_{0\ldots i + k}, O_{0\ldots j + k})| = |\text{LCS}(R_{0\ldots i^{\prime } +k^{\prime }}, O_{0\ldots j^{\prime } + k^{\prime }})| - 1$.

The edges are labeled with the replacement: $i + k + 1 : i^{\prime } + k^{\prime } / O_{j + k + 1 \ldots j^{\prime } + k^{\prime } - 1}$. For example in Fig. 5 there is an edge between nodes $(5, 5, 3)$ and $(7, 8, 1)$ labeled $7\!:\!7\, /\, \mathtt {T}$ with $k = 1$ and $k^{\prime } = 0$. Note that we do not only compress nodes; we also compress edges when all minimal replacements between two nodes are present, e.g. $1\!:\!2\, /\, \mathtt {T}$ represents the two different alignments [$1\!:\!1\, /\, \mathtt {T}$, $1\!:\!2\, /\, \mathtt {}$] and [$1\!:\!2\, /\, \mathtt {}$, $2\!:\!2\, /\, \mathtt {T}$].

We can systematically and efficiently construct the graph using the LCS-nodes data structure. Processing the elements in the LCS nodes is based on their index from high to low. We check the elements at an indexed LCS position from right to left, as they are conveniently ordered as a direct consequence of when they were discovered during the alignment. For an element with length $\ell$ at index $p$, we check the conditions with all other elements at indices in ${p, p-1, \ldots , p - \ell }$. When the conditions are met, the corresponding edges are added to the graph. The current element is then discarded from the LCS-nodes data structure. In situations when there is no match at the beginning and/or at the end of the sequences, a respective source and/or sink node with an $\epsilon$ match is added to the graph, so that also these graphs have a unique source and sink node.

Not all processed matches are part of the ultimate alignments. Because of the processing order these elements can be easily recognized, as they have no path to the sink node, and are therefore not added to the graph. For example element $(3, 1, 1)$ from Fig. 3 is not part of any LCS alignment. In general, these elements are also computed by the ONP algorithm (see Fig. 2).

#### Splitting matches

**Figure 6.**
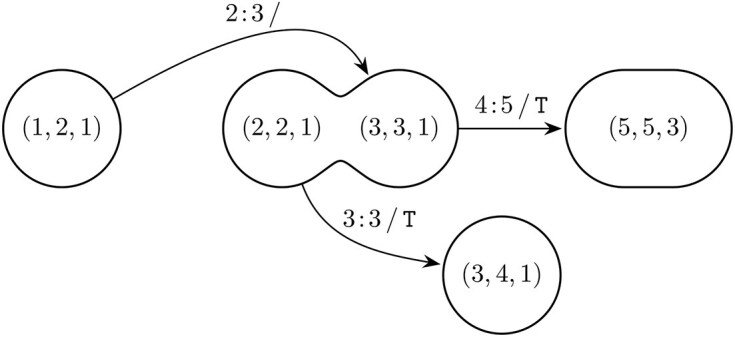
The immediate neighborhood of node $(2, 2, 2)$ from Fig. 5. Node $(2, 2, 2)$ is split into two nodes $(2, 2, 1)$ and $(3, 3, 1)$ in order to avoid path [$2\!:\!3\, /\, \mathtt {}$, $3\!:\!3\, /\, \mathtt {T}$].

As a consequence of compressing the consecutive matches, we run the risk of introducing “inverse” paths (alignments) that are not present in the classic LCS-graph. These inversions are caused by incoming edges that enter compressed nodes “beyond” outgoing edges. In Fig. 6 we illustrate the situation occurring with element $(2, 2, 2)$ from the cLCS-graph in Fig. 3.

Element $(2, 2, 2)$ represents two consecutive matches, present as separate nodes $(2, 2)$ and $(3, 3)$ in the LCS-graph. Without countermeasures, the path [$2\!:\!3\, /\, \mathtt {}$, $3\!:\!3\, /\, \mathtt {T}$] would be present. However, this path does not exist in the LCS-graph because of the explicit (unlabeled) edge between $(2, 2)$ and $(3, 3)$. To remedy this situation, we create a new node by splitting the conflict-inducing node immediately before the conflicting incoming edge and connect these two nodes with an unlabeled ($\lambda$) edge. For the remainder of the calculation only the first (newly created) node needs to be considered.

### Variant description extraction

The cLCS-graph introduced above contains all relevant changes for a genetic variant and could therefore be considered a complete representation of that variant, often corresponding to multiple variant descriptions. In contrast, in the domain one often needs to choose a single textual variant description for practical purposes. In order to obtain a variant description from a cLCS-graph, we define the problem of extraction: to calculate a route from source to sink allowing us to use both existing edges and additional shortcut replacements between two nodes that are not directly connected, where the shortcuts do not introduce cycles in the graph. Shortcuts enable summarization of (potentially) complex parts of the graph, but do not represent an LCS alignment.

**Figure 7.**
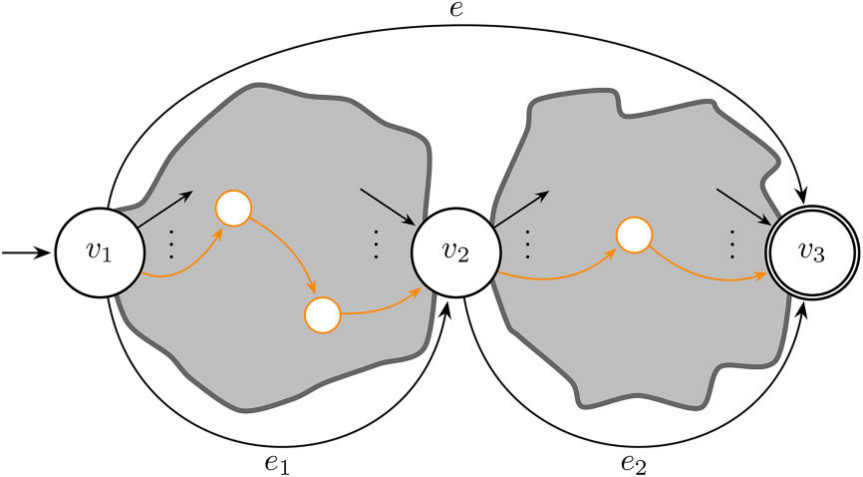
A schematic view of an instance of a cLCS-graph with the supremal variant, all-encompassing single replacement $e$. All paths go through nodes $v_1$, $v_2$ , and $v_3$, partitioning the graph in local supremal parts described by replacements $e_1$ and $e_2$. The highlighted path is the canonical path.

Within the domain, the ultimate goal regarding variant descriptions, is to determine a single variant description out of the numerous ways of describing that variant, often beyond considering only LCS alignments. In this section we present three alternative extraction methods specifically tailored toward yielding descriptions with relevance in the domain. Figure 7 gives a schematic overview of the three methods and their relationships: (i) the *supremal variant*, which is especially suited for describing small deletion-insertions analogous to SPDI with VOCA normalization. It is a single unique all-encompassing replacement ($e$ in Fig. 7); (ii) the *local supremal variant* that naturally extends the supremal variant to a version that caters for allele descriptions (multiple small deletion-insertions *in cis*) ($e_1$ and $e_2$ in Fig. 7); and (iii) the *canonical variant*, which is specifically geared toward variant reporting in the domain, e.g. using HGVS (the highlighted path in Fig. 7). The NCBI uses the term “Canonical SPDI” for similar purposes, but note that these are not necessarily the same.

#### Supremal variants

Every variant can be described by the complete deletion of $R$ and the subsequent insertion of $O$. Stretching that argument to the extreme, the variant can thus also be described by only $O$ (with an empty $R$). For small variants on large sequences this is generally not practical.

The supremal variant is a similar, but generally more compact, description of a single replacement that captures all relevant changes. It is also used for calculating the relation between two variants as introduced in [22]. In this paper we restrict ourselves to the changes within the supremal variant for determining variant descriptions. Using the cLCS-graph, the supremal variant is constructed as a single replacement between the source and sink nodes, by taking the position of the first outgoing edge of the source node and the position of the last incoming edge of the sink node. For the example in Fig. 5, the supremal variant is $1\!:\!8\, /\, \mathtt {TCTTACTT}$.

In practice, variants are small changes with regard to a large reference sequence, leading to many consecutive matches in both the source and sink nodes. Most of these matches are outside of the supremal variant. For convenience we trim the source and sink nodes to only include the matches that are inside the supremal variant. For the example in Fig. 5 this leads to relabeling of the source node from $(0, 0, 1)$ to $(1, 1, 0)$. The sink remains unchanged as the matching T at $(7, 8)$ is inside the supremal variant. Note that after trimming, the supremal variant can be derived directly from the source and the sink node labels without looking at the edges.

Conversely, a supremal variant description is the minimally required input for constructing the cLCS-graph. In practice this makes the supremal variant description a good candidate for storing variants. VOCA used in SPDI and VRS attempts to achieve a similar goal by enlarging the variant. However, this often yields a smaller deletion-insertion than the supremal variant.

Even without initial access to the supremal variant, it often pays off to repeatedly estimate the supremal variant when constructing the cLCS-graph and checking whether the estimation is correct, by determining if matches are trimmed at both the source and the sink. If not, the estimate should be enlarged.

#### Local supremal variants

In the domain it becomes more common to look at combinations of multiple changes. These combinations on the same molecule are said to be phased, *in cis* or part of the same allele and can be written down as phase sets, e.g. in VCF, or allele descriptions, e.g. in HGVS.

The technique from the previous section properly calculates the supremal variant for alleles too. Despite being technically correct, in many cases it is not a convenient way to describe such combinations of changes as they can result in very large deletion-insertions. Here we use a specific property of the cLCS-graph to come to more meaningful descriptions.

In Fig. 5, we identify three nodes in the cLCS-graph that are part of every path from the source node to the sink node: $(0, 0, 1)$, $(5, 5, 3)$ , and $(7, 8, 1)$. All paths have at least one common match in each of these nodes, with the possible exception of zero-length source and sink nodes. We argue to partition the graph using these common matches.

The nodes we have identified are called *dominators* [27] of the sink node in the field of control-flow analysis [28]. Note that these dominators should not be confused with domination used in the construction of the cLCS-graph. Analogous to the definition of dominators, we define the set of *post-dominators* of a node $n$ as:


(2) \begin{eqnarray*}
\mathrm{PostDom}(n) = \left\lbrace \begin{array}{@{}l@{\quad }l@{}}\left\lbrace n \right\rbrace & \mathrm{if}\, n = n_0, \\\left\lbrace n \right\rbrace \cup \bigcap _{s \in \mathrm{successors}(n)} \mathrm{PostDom}(s) & \mathrm{otherwise}. \end{array}\right.
\end{eqnarray*}

As our graph has a single sink node, finding the post-dominators of the source node results in the same nodes as finding the dominators of the sink node. In implementations where only outgoing edges are stored, post-dominators can be calculated more efficiently.

An efficient algorithm applies Equation 2 for each node $n$ in a single post-order traversal, while additionally keeping track of the maximal position for each incoming edge per node. The resulting set of post-dominators for the source node $\lbrace (0, 0, 1), (5, 5, 3), (7, 8, 1)\rbrace$ indeed consists of the nodes that are part of every path in the graph. Making use of the maximal position of the incoming edges for each of these nodes and the minimal positions of their outgoing edges, we construct the replacements between these nodes analogous to the construction of the supremal variant. This results in the local supremal variant with replacements $1\!:\!5\, /\, \mathtt {TCTT}$ and $7\!:\!8\, /\, \mathtt {TT}$. In HGVS this is described as [2_5delinsTCTT;8delinsTT].

**Figure 8.**
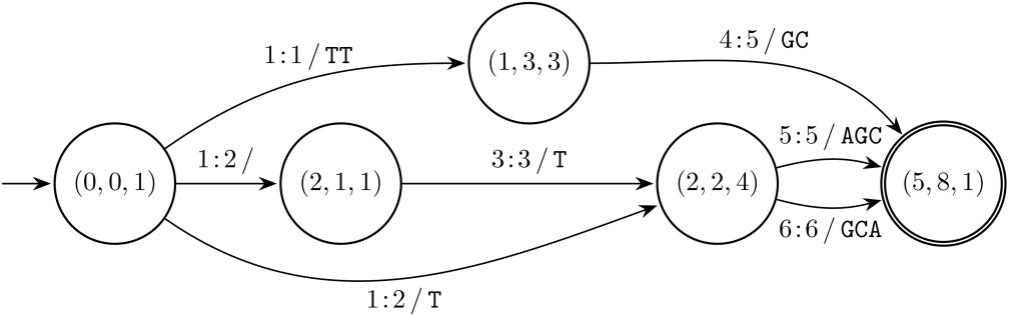
The cLCS-graph of [$1\!:\!1\, /\, \mathtt {TT}$, $4\!:\!5\, /\, \mathtt {GC}$] with $R = \mathtt {CATATATCG}$. Other than the source and the sink nodes, there is no node through which all paths traverse.

Perhaps counterintuitively, an allele description composed from more variants that are supremal variants to a reference, does not necessarily lead to a local supremal variant with those variants as parts. For example combining supremal variants $1\!:\!1\, /\, \mathtt {TT}$ and $4\!:\!5\, /\, \mathtt {GC}$, with $R = \mathtt {CATATATCG}$, does not yield the local supremal variant [$1\!:\!1\, /\, \mathtt {TT}$, $4\!:\!5\, /\, \mathtt {GC}$], but a single replacement $1\!:\!6\, /\, \mathtt {TTATAGCA}$ as (local) supremal variant shown in Fig. 8, emphasizing the need for an extraction method specifically for variants that consist of multiple parts.

#### Choosing a canonical variant

So far we have used graph properties to find (local) supremal variants, which generally lead to non-minimal coarse-grained replacements. However, in the domain the variant descriptions are often more fine-grained. We introduce a third extraction method based on the well-known shortest path problem. Intuitively, when presented with a choice of paths through the graph, shorter paths lead to more concise descriptions. We consider the subgraph of the cLCS-graph consisting of only the shortest paths. All edges have weight 1 except for unlabeled ($\lambda$) edges, which have weight 0. For the example in Fig. 5, when we consider the part of the graph between nodes $(0, 0, 1)$ and $(5, 5, 3)$, there is a unique shortest path of weight 2 with edges $1\!:\!2\, /\, \mathtt {T}$ and $4\!:\!5\, /\, \mathtt {T}$.

If there is no unique shortest path, we employ the extraction method from the previous section on the shortest paths subgraph. In Fig. 5 there is no unique shortest path in the part of the graph between nodes $(5, 5, 3)$ and $(7, 8, 1)$. The two edges, with weight 1, $7\!:\!7\, /\, \mathtt {T}$ and $8\!:\!8\, /\, \mathtt {T}$ result in the replacement $7\!:\!8\, /\, \mathtt {TT}$. Here, these parallel edges indicate the presence of a repeat unit expansion.

#### Constructing HGVS descriptions

The extraction of the canonical variant results in a series of replacements. In HGVS more domain-specific constructs exist. Here we translate our replacements to HGVS descriptions. If for a replacement the deleted or inserted part is empty, the HGVS counterpart is an ins or del, respectively, e.g. in Fig. 5, $1\!:\!1\, /\, \mathtt {T}$ translates to 1_2insT and $4\!:\!5\, /\, \mathtt {}$ translates to 5del. A replacement where both the deleted and inserted parts have length 1 is described in HGVS as a substitution [also called a single nucleotide variant (SNV) in the domain], e.g. in Fig. 5, $2\!:\!3\, /\, \mathtt {T}$ translates to 3C>T.

For the remaining replacements, we check if the replacement is a proper repeat expansion or contraction using a single repeat unit by using the Longest Prefix Suffix preprocessing step from the Knuth–Morris–Pratt algorithm [29]. The identified repeat cases are then described using the HGVS bracketed repeat syntax, with an exception for a repeat expansion from 1 to 2 units, which is described as a duplication, e.g. in Fig. 5, $7\!:\!8\, /\, \mathtt {TT}$ translates to 8dup. Alternatively, if the inserted part of the replacement is the reverse complement of the deleted part, we describe this as an HGVS inversion. Otherwise the replacement is described using the HGVS delins construct. The HGVS description for the example from Fig. 5 is [2C>T;5G>T;8dup].

## Results

In order to assess the impact of our method on prevailing variant descriptions, we compare our extracted HGVS descriptions to descriptions in dbSNP [8] Build 156. The dbSNP database is a popular public resource containing human single nucleotide variations, microsatellites, and small-scale insertions and deletions, generally considered the definitive source for variant descriptions. For all variants on genomic reference sequence NC_000001.11 (chromosome 1 of GRCh38), we retrieve both the SPDI and HGVS descriptions. The SPDI descriptions are used as input to construct the sequences to apply our method to. Our generated HGVS descriptions are then compared to the respective HGVS descriptions from dbSNP. For an indication of performance, our Python implementation is able to compare ~40 variants per second per core on modern hardware.

Table 1 shows the results of the comparison of our descriptions using the method from the previous section with the descriptions in dbSNP. We skipped 18 555 entries because of uninterpretable HGVS descriptions in dbSNP. We have reported those to the maintainers of the database and they will hopefully get corrected in a future release. Additionally, because of the exact nature of our method, we skipped 668 entries with ambiguous nucleotide symbols such as N.

**Table 1.** The categorized counts of the textual comparisons between the HGVS entries in dbSNP and our generated HGVS descriptions

**Identical**	
Substitutions (SNVs)	$91\;487\;743$
Indels	$5\;374\;091$
Repeats	$1\;278\;940$
Inversions	$3374$
**Different**	
Mononucleotide repeats	$3\;434\;553$
Mixed repeats	$317\;578$
Inserted sequence repeats	$154\;866$
Allele descriptions	$10\;310$
Inversions	$26$
**Other**	
Inserted sequence repeats	$18\;555$
Ambiguous nucleotides	$668$
Total	$102\;080\;704$

There are 98 144 148 identical descriptions, which include all 91 487 743 SNVs present in the database (e.g. 175292543T>C). The remaining identical descriptions are 6 656 405 out of the 10 573 738 non-SNVs. These additional identical descriptions include deletions, e.g. 206841458del; insertions, e.g. 205259305_205259306insAG; simple multinucleotide repeats, e.g. 93576231_93576234AC[3]; and inversions, e.g. 195156503_195156504inv. Although the simple multinucleotide repeats slightly differ in syntax from the entries in the database, most likely because of changed HGVS recommendations regarding repeats, they are semantically identical.

Shifting our attention to the differences, for a total of 3 917 333 variants, we provide a different description than the entries in dbSNP. By far the largest fraction of differences consists of mononucleotide repeats. For a representative example, dbSNP describes a duplication 175145557_175145575dup, while we describe a repeat expansion 175145553_175145575A[42]. Similarly, dbSNP describes mononucleotide repeat contractions as a deletion. When a repeat expansion cannot be expressed as a duplication (too few repeat units present in the reference), it is described in dbSNP as an insertion instead. This is surprising, as multinucleotide repeats are actually described as repeat expansions or contractions in dbSNP. Furthermore, the normalized SPDI representation does cover all the repeat units in the reference. Also, in [30] an explicit example is given of a mononucleotide repeat expansion described as such in HGVS. We prefer the repeat notation for all these cases.

Central to the main message of this paper, 10 310 variants that are described as a deletion-insertion in dbSNP are described as an allele by us. For example 148693179_148693189delinsGGAAATAAAAC is described by us as [148693179A>G;148693189G>C]. Interestingly, both variants 148693179A>G and 148693189G>C also appear individually in the database.

While most of the inversions turn up identical, a small number of inversions in dbSNP are described by our method as an allele, e.g. 221541589_221541591inv versus [221541588_221541589insC;221541591del].

Besides describing contractions or expansions of repeat units in the reference sequence, HGVS also prescribes a repeat-like notation to compress the inserted sequence. In this experiment there are 154 866 inserted repeated sequences in dbSNP, also including mononucleotide repeats, that are described by our method as a repeated insertion, e.g. 149733078delinsTGTTTTGTTTTGTTTTGTTT versus 149733078delinsTGTTT[4].

The last category to address consists of mixed repeats. In the dataset from dbSNP there are 317 578 entries that describe variants composed of multiple repeat units and their respective repeat count, e.g. 155164866_155164881T[23]GTTTTTTTTT[2]T[14]. Our method does not generate such descriptions, as we currently only consider single repeat units. The above example we describe as 155164881_155164882insTTTTTTTGTTTTTTTTTGTTTTTTTTTTTTTTTTTTTTTTT. It is worth noting that all mixed repeat entries in this experiment are effectively insertions and never contractions.

Intrigued by these dbSNP entries and to further understand these mixed repeats, we queried the NCBI Variation Service [8]. Surprisingly, 247 610 entries could not be interpreted by the NCBI at all. Additionally, 9491 entries resulted in a warning, with only a partial answer returned. We identified that rewriting the mixed repeat into a deletion-insertion, where the inserted sequence consists of the individual repeat units and their counts, results in a variant that is equivalent to the input SPDI. The example is therefore rewritten as 155164866_155164881delins[T[23]; GTTTTTTTTT[2];T[14]]. However, HGVS states that all repeat units need to be present in the reference sequence. Therefore, a more conformant description is [155164866_155164881T[23];155164881_ 155164882ins[GTTTTTTTTT[2];T[14]]] as the trailing repeat units GTTTTTTTTT and T are not present in the reference at this location.

## Discussion

The HGVS descriptions produced with our method are concordant with all SNVs and most simple insertion and deletion entries in dbSNP. For the remaining (non-trivial) entries our method often yields insightful results.

Built on the premise that considering all LCS alignments is useful or even required for meaningful extraction of variant descriptions, the cLCS-graph as variant representation provides additional ways to analyze variants and could itself be considered an alternative for textual descriptions.

Rigorous methods are required to effectively deal with ever more complex variants both at scale and for correctness. We argue that the focus of standardization committees, e.g. for HGVS, should be more on comprehensibility instead of (manual) constructability.

In this section we explore some interesting examples for classes of variants as well as limitations of our method.

### Compound variant descriptions

While dbSNP is not intended for allele descriptions, we found that there are entries described as a single deletion-insertion that consist of multiple other smaller variants. Often these smaller variants also exist independently in dbSNP.

Conversely, in some cases, our method results in allele descriptions that consist of many variants, where the relatively short deletion-insertion description is arguably more practical, e.g. 51172616_51172630delinsACACC versus [51172616G>A;51172617_51172618insA;51172620 _51172630del]. At the same time the value of our method is illustrated by the fact that the substitution in our allele description is also present in dbSNP individually. To decide a priori (or even a posteriori) whether the allele description has added benefit is challenging, for instance [119251105del;119251107del;119251109del; 119251111del;119251113del] versus 119251105_119251113delinsAAAA. While the deletion-insertion description is concise, the allele description clearly communicates that this variant only consists of deletions. Note that when the deletion-insertion notation is preferred, a more concise way to describe this variant is 119251105_119251113delinsA[4]. Our earlier method [16] attempted to make the decision of reporting a variant as a deletion-insertion when the description became too complex. We do not do that now, because we want to make the reference and variant structure leading instead of the simplicity of the HGVS syntax.

### Atomicity of variants

The individual parts of the local supremal partitioning are independent and suggest a natural boundary for allele construction. Determining whether a variant should be described as one or more elements has been a long-debated issue within the HGVS Variant Nomenclature Committee. A community consultation [31] has been created in an attempt to resolve the discussion. As the title “var distance” already suggests, it proposes “two variants that are separated by fewer than two intervening nucleotides (that is, not including the variants themselves) should be described as a single ‘delins’ variant.” As there is no notion of distance between two variants, that rules out using distance as a metric for variant separation. When the local supremal variant consists of multiple parts, we argue one should be hesitant to describe such variant in a different partitioning. The independence of the parts of a local supremal variant cannot only be used for the sake of descriptions, but also has practical implications. The parts can be stored and processed individually and therefore allow for optimization.

### Database considerations

Variants are often stored in and retrieved from databases for both clinical and research purposes. In the case of dbSNP, this is in the form of SPDI, but in many cases HGVS variant descriptions are used. Using HGVS variant descriptions for retrieval has the obvious disadvantage of missing findings because of alternative notations. The entries in dbSNP are internally represented as SPDI, which are VOCA normalized. While their region-based approach resembles our approach, it does not include some of the context that is required for a complete variant representation.

**Figure 9.**
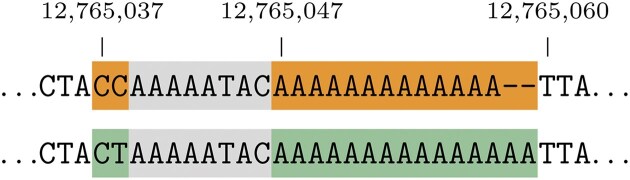
A visualization of 12765038_12765046delinsTAAAAATACAA from dbSNP. Shown is a slice of the reference (top) and the observed sequence (bottom). Deletions are highlighted in orange and insertions in green. The supremal variant is the whole highlighted area, including the gray, where the latter is not part of the local supremal variant [12765037_12765038delinsCT;12765047_12765059delinsAAAAAAAAAAAAAAA].

The differences are that VOCA operates on the input variant description directly, while the input variant description for us is merely a way to construct the observed sequence, and that VOCA is not practical for allele descriptions. This is illustrated in Fig. 9. VOCA identified the region 12 765 038–12 765 046 as relevant, which includes the substitution of the C to T on the start position until the end position, after which it includes the two additional As. We on the other hand, using the supremal variant, identify the region 12 765 037–12 765 059 as relevant. It starts one base to the left as that C can also be removed, and ends at the last of the 13 repeating As. The latter enables us to actually detect it as a repeat expansion. Again, by calculating the local supremal variant, we identified two independent parts of the variant, which are also both individually present in the database.

### Complex repeats

In addition to syntactic differences in repeat notation mentioned earlier, the mixed repeat category deserves further elaboration. The problem of complex repeat detection in a single string has been studied before, e.g. in [32]. The application in the context of the description of genetic variants is still an open problem. The fact that the NCBI Variation Service does not accept a substantial share of the mixed repeat entries in dbSNP from the experiments also indicates that this is unexplored territory. As a consequence, our method currently does not result in mixed repeat descriptions. We do, however, see value in being able to report on this type of variant. While the dbSNP mixed repeats clearly do not follow the HGVS recommendations, in general the HGVS nomenclature is currently underspecified with regard to mixed repeats.

**Figure 10.**
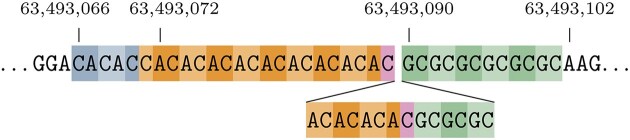
A visualization of the mixed repeat 63493089CA[4]CG[3]C[1] on a slice of the reference sequence, effectively describing an insertion of $4\times \mathtt {AC}$ highlighted in orange and $3\times \mathtt {GC}$ highlighted in green described in HGVS as 63493089_63493090ins[AC[4];GC[3]]. Both the AC and GC also occur in the reference. Note that these patterns partially overlap as highlighted in purple. The supremal variant identifies an additional adjacent relevant occurrence of an unchanged CA repeat highlighted in blue.

We turn to Fig. 10 for an example to guide the mixed repeats discussion. The dbSNP HGVS description 63493089CA[4]CG[3]C[1] has a corresponding SPDI entry 63493088:C:CACACACACGCGCGC. This entry is effectively described as an insertion, and the VOCA region does not span the existing repeat units in the reference. It therefore begs the question if the reference actually plays a role here, or if the repeat notation is based on the inserted sequence only. The supremal variant includes all the repeat units and the adjacent CA repeat starting from 63 493 066. The description can start either at 63 493 071 with the CA and CG rotation or at 63 493 072 with the AC and GC rotation. With that context, we can expand both the AC/CA and CG/GC repeat units to 13 and 9, respectively. HGVS dictates the latter because of its shifting rule: 63493072_63493089AC[13] and 63493090_63493101GC[9]. Combining the two into the current mixed repeat format results in 63493072_63493101AC[13]GC[9], conveniently getting rid of the C[1] from the original entry as there is no such repeat in the reference. The disadvantage of this format is that we cannot determine how many repeat units there were originally in the reference. An alternative way to address that issue is to describe this variant as allele [63493072_63493089AC[13];63493090_ 63493101GC[9]]. Note that patterns AC and CG partially overlap, which in general leads to multiple equivalent descriptions.

### Biological features

With our current methods, inversions are not detected natively. However, when the shortest path to describe an inversion is a single replacement, we attempt to see if that replacement can also be expressed as an inversion. For the concrete case of our experiment on dbSNP, we are able to trivially identify all inversions when applying this strategy to the supremal variant. Unfortunately, when an inversion is more aptly represented as an allele description it cannot be identified without additional efforts. Our former extraction method [16] performs an alignment against both the forward and reverse complement of a reference sequence. A similar technique might be applied to the alignment described in this paper.

So far we have reported only on chromosomal reference sequences, yielding genomic variant descriptions in HGVS. In practice, variants in HGVS are often reported relative to coding transcript references. While the variant descriptions resulting from our methods can be mapped to transcript references (using, for instance, the Mutalyzer tool suite [18]), our extraction methods do not take the features, i.e. introns and exons, of these transcripts into account. This implies that for variants on the exon-intron boundary, equivalent variant descriptions on genomic level could have different effects on the transcript.

### Performance challenges

The nature of the alignment method can lead to (sometimes counterintuitively) large graphs. This presents performance challenges for larger variants, where memory usage grows significantly. This is especially true for large deletions, which have a large number of paths, while having compact descriptions. While the current Python implementation is adequate to perform a single experiment on chromosomal scale, for routine whole genome sequencing analysis this implementation could be considered too slow. For these situations a reimplementation in a more performance-oriented language might be in order.

### Conclusions

In this paper we addressed the problem of finding suitable representations for genetic variants and subsequently choosing particular descriptions for each variant. Beyond the crucial property that variant descriptions need to be unambiguously interpretable, there is a clear need for directions on how to describe them in various scenarios. The introduced cLCS-graph data structure is a complete representation of a variant. It enables the three extraction methods presented in this paper that address the needs for usage of variant descriptions ranging from clinical reporting to databases. Experiments clearly show the benefits of our methods.

Together with the comparative capabilities of the variant algebra, one is ultimately free in how to choose a variant description while still being able to relate them to other notations of the same variant or other variants altogether. Although there is not always clear added benefit for writing variants as alleles, our method does enable this through local supremal variant extraction. Besides practical value as demonstrated in the experiments, our deterministic extraction methods also support rational choices for Variant Nomenclature Standard processes.

### Future work

Our methods are currently agnostic to genomic features and could be enriched to enable exon-intron-aware variant extraction. Inversions and transpositions could be enabled analogous to the way inversions are dealt with in [16]. Earlier experiments showed that there are many variants in dbSNP that have a non-disjoint relation with one or more other variants in the database. By extending dbSNP with our concepts it would enable richer query possibilities and allele descriptions.

## Data Availability

A Python implementation is available at https://github.com/mutalyzer/algebra/tree/v1.5.2 with a snapshot deposited at https://doi.org/10.5281/zenodo.17241368 and integrated in a web interface at https://test.mutalyzer.nl/normalizer.
